# Total versus conventional laparoscopic cyst excision and Roux-en-Y hepaticojejunostomy in children with choledochal cysts: a case–control study

**DOI:** 10.1186/s12893-020-00906-5

**Published:** 2020-10-17

**Authors:** Fei Liu, Xiaogang Xu, Menglong Lan, Boyuan Tao, Le Li, Qiang Wu, Chengwei Chai, Jixiao Zeng

**Affiliations:** Department of Pediatric Surgery, Guangzhou Institute of Pediatrics, Guangzhou Women and Children’s Medical Center, Guangzhou Medical University, 9 Jinsui Road, Guangzhou, 510623 Guangdong China

**Keywords:** Total laparoscopic, Choledochal cyst, Children, Congenital

## Abstract

**Background:**

To compare the efficacy of total and conventional laparoscopic hepaticojejunostomy (TLH and CLH) in children with choledochal cysts (CDCs).

**Methods:**

Data from patients undergoing TLH and CLH between August 2017 and December 2018 were retrospectively analyzed. Intraoperative blood loss, time for jejunum-cojejunum anastomosis, time to oral intake, postoperative hospital stay, hospitalization expenses, and postoperative complications were compared.

**Results:**

All 55 patients (TLH = 30, CLH = 25) were successfully treated without conversion to open surgery. In the TLH and CLH groups, the time to oral intake was 3.57 ± 0.19 d and 4.56 ± 0.27 d, respectively (*t* = 3.07, *P* < 0.01), the postoperative hospital stay was 5.50 ± 0.28 d and 7.00 ± 0.74 d (*t* = 2.03, *P* < 0.05), and the hospitalization expenses were CNY 40,085 ± 2447 and CNY 26,084 ± 2776 (*t* = 3.79, *P* < 0.001). There were no significant differences in intraoperative blood loss (9.57 ± 3.28 ml vs 8.2 ± 1.13 ml, *t* = 0.37, *P* = 0.72) or time for jejunum-cojejunum anastomosis (80.5 ± 2.46 min vs 75.00 ± 2.04 min, *t* = 1.68, *P* = 0.10). The median follow-up periods of the TLH and CLH groups were 17 and 16 months, respectively. Overall complication rates were comparable between the two groups (10% vs 8%, *χ*^2^ = 0.07, *P* = 0.79).

**Conclusions:**

TLH in children with CDCs has the advantages of rapid gastrointestinal functional recovery and a short hospitalization. However, hospitalization is relatively expensive.

## Background

Choledochal cysts (CDCs) are a rare congenital biliary malformation. The incidence of CDCs in Western countries ranges from 1/50,000 to 1/200,000, while that in Japan is approximately 1/13,000, with a male-to-female ratio of 1:4 [1–3]. Total cyst excision with Roux-en-Y hepaticoenterostomy has become the standard procedure [4]. In 1995, Farello et al. [5] reported for the first time the laparoscopic-assisted treatment of CDCs. Currently, the laparoscopic treatment of CDCs has been widely accepted by surgeons and children due to its advantages of a small incision, less trauma, less pain and a quick recovery [6, 7]. The gradual improvement of laparoscopic instruments and surgical techniques has made it possible to treat CDCs in children by total laparoscopy. However, considering the limited space of the abdominal cavity, the difficulty of the operation and the high cost of hospitalization, it has not been widely performed [8]. From August 2017 to December 2018, we compared the efficacy of total laparoscopic hepaticojejunostomy (TLH) and conventional laparoscopic hepaticojejunostomy (CLH) in children with CDCs.

## Methods

We retrospectively analyzed the data of 30 children who underwent TLH and 25 children who underwent CLH from August 2017 to December 2018. All patients were evaluated by ultrasonography, computed tomography (CT), or magnetic resonance cholangiopancreatography (MRCP) before the operation. Considering the limited abdominal cavity space of a child, we set the inclusion criteria for the TLH group as children > 1 year old with CDCs. The exclusion criteria were as follows: (a) children < 1 year old, (b) children with a history of upper abdominal surgery, (c) children who experienced uncontrolled acute cholangitis, (d) children who suffered from cardiovascular or other diseases that contraindicate total laparoscopic surgery, or (e) children whose parents did not accept total laparoscopic surgery.

Patients with no following preconditions were included in the CLH group: (a) upper abdominal surgery history, (b) uncontrolled acute cholangitis, (c) cardiovascular or other diseases that contraindicate laparoscopic surgery, and (d) parents who did not accept laparoscopic surgery. Ethical approval from the Institutional Review Board of Guangzhou Women and Children’s Medical Center and written parental consent were obtained. The research was conducted in compliance with the World Medical Association Declaration of Helsinki.

### Operative techniques

#### Total laparoscopic CDC excision and Roux-en-Y hepaticojejunostomy

Supine position was employed. Different carbon pneumoperitoneum pressures were established according to different ages: age ≤ 1 year, 6–8 mmHg; 1–3 years: 8–10 mmHg; and > 3 years: 10–12 mmHg, respectively [6]. A 5-mm port was built via a transumbilical incision, through which a 5-mm 30° laparoscope was introduced. Under the laparoscopic guidance another three ports were built with the second and third ones below the costal margin in the right and left upper quadrants and the fourth one below the second.

**Fig.1:**
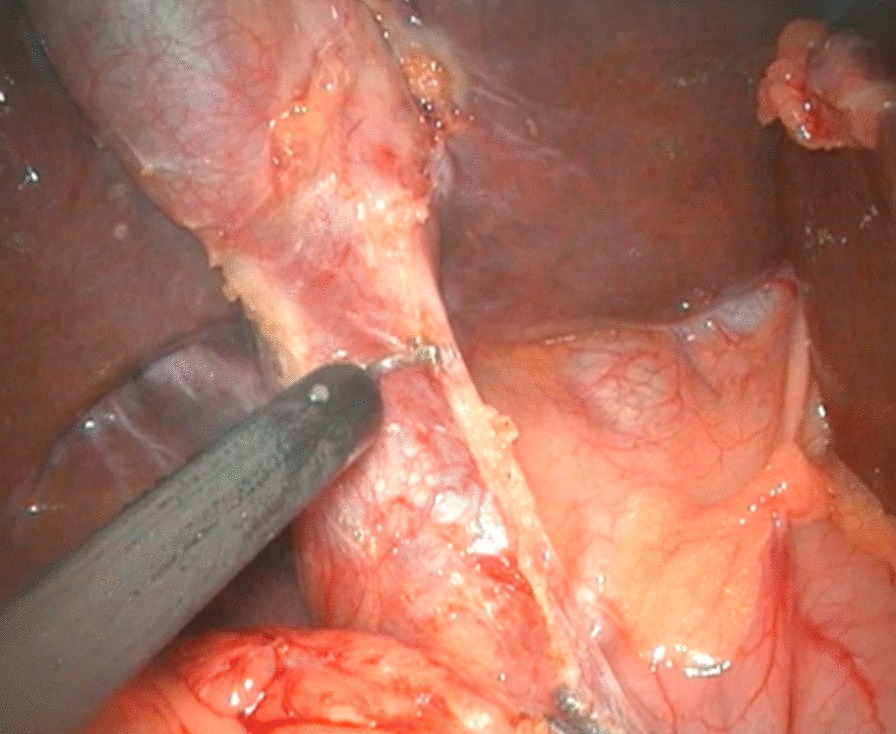
Separation of anterior wall of choledochal cyst

**Fig.2:**
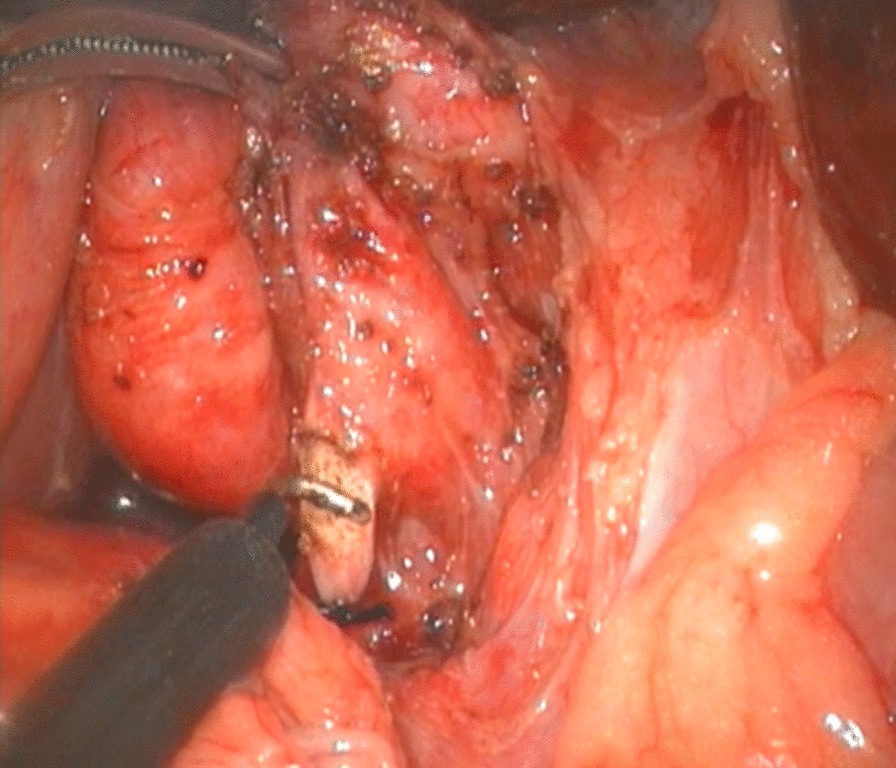
Ligating and excising distal end of common bile duct

**Fig.3:**
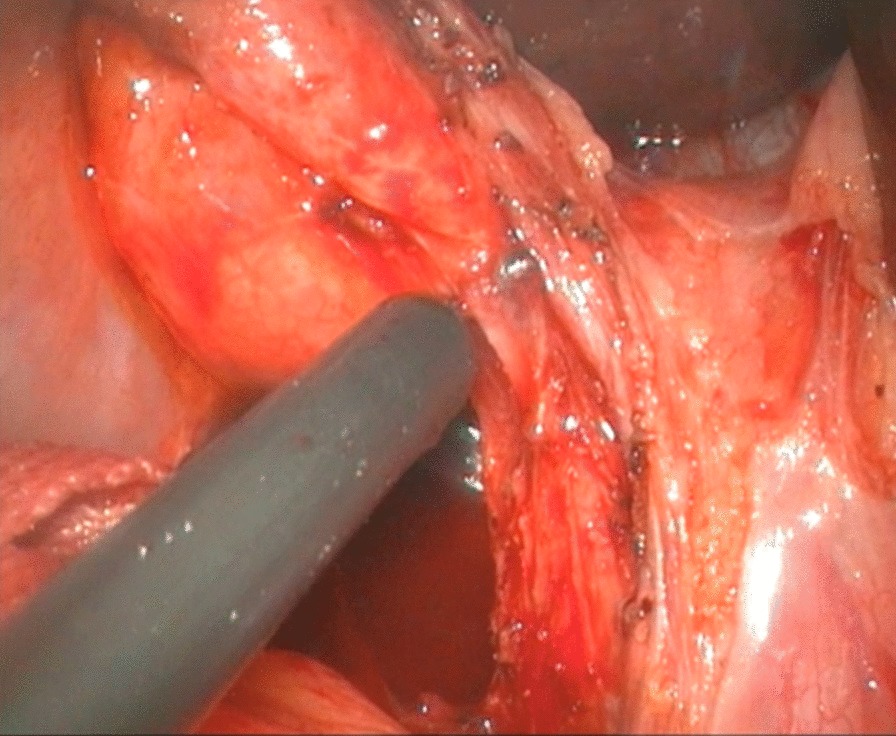
Separation of posterior wall of choledochal cyst

To expose the hepatic hilum, a 2–0 hitch stitch was used to pass through the anterior abdominal wall and falciform ligamentum. A monopolar electrocautery instrument was used to initiate the cyst dissection anteriorly and continue it distally to the posterior duodenal wall (Fig. 1). The cleavage between the cyst and the pancreas could be identified clearly when a rim of the lower portion of the cyst was held cephalad by the operator and the duodenum was pulled caudal by the assistant. Then started the duct system examination for stone, debris and ductal stenosis after transversely opening the anterior wall of the cyst, which were specifically efficient under the laparoscopically magnified view. Saline irrigation of the duct was conducted to clear out the stones and debris if found. Laparoscopic ductoplasty was performed if stenosis was identified. Thereafter dissected the full thickness of the cyst as close as possible to its wall using the cautery instrument until the narrow portion to the cyst appeared. Finally got the narrow portion ligated securely with a 4–0 nonabsorbable suture and transected (Fig. 2). Subsequently, the distal part of the cyst was dissected close to the posterior wall of the cyst (Fig. 3) to avoid injury to the portal vein. The cyst was completely excised close to the hepatic hilum at the junction between the normal common hepatic duct and the dilated cystic wall.

**Fig.4:**
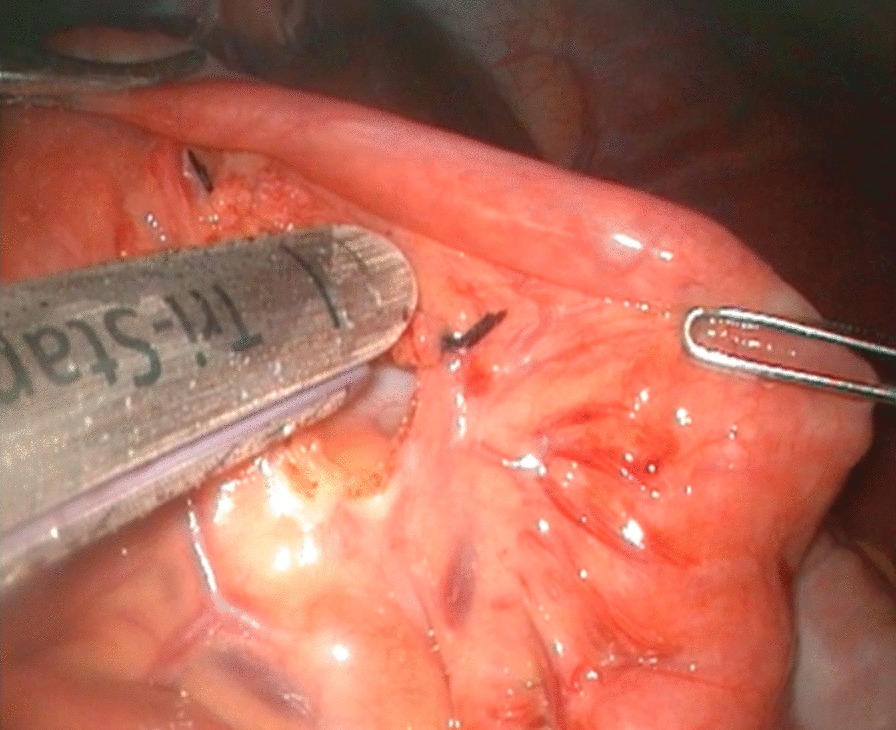
Excising proximal jejunum with Endo-GIA tri-staple

**Fig.5:**
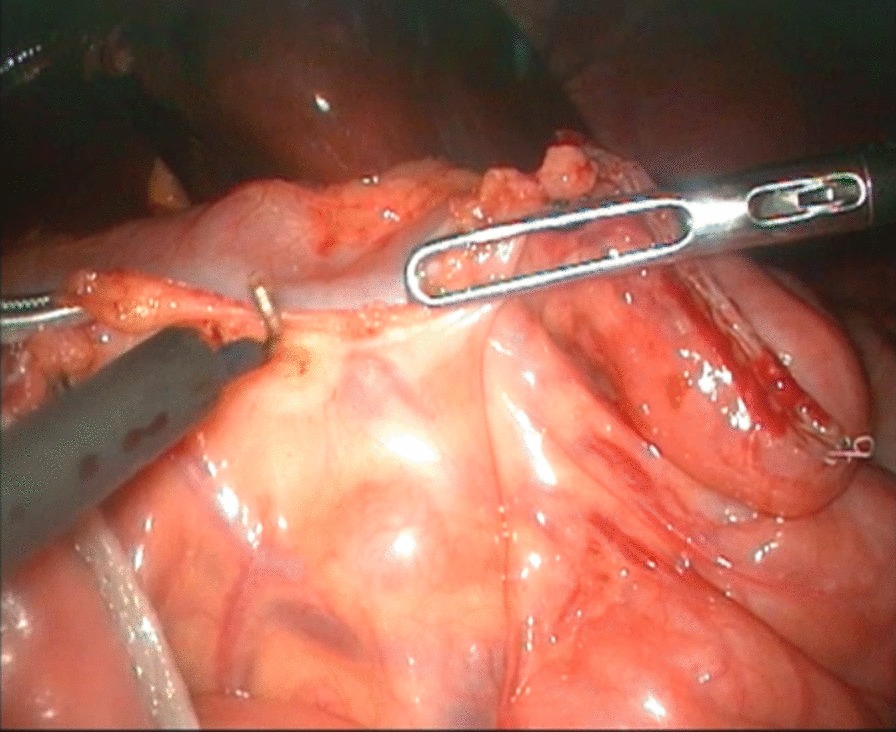
Fully releasing jejunal mesentery

**Fig.6:**
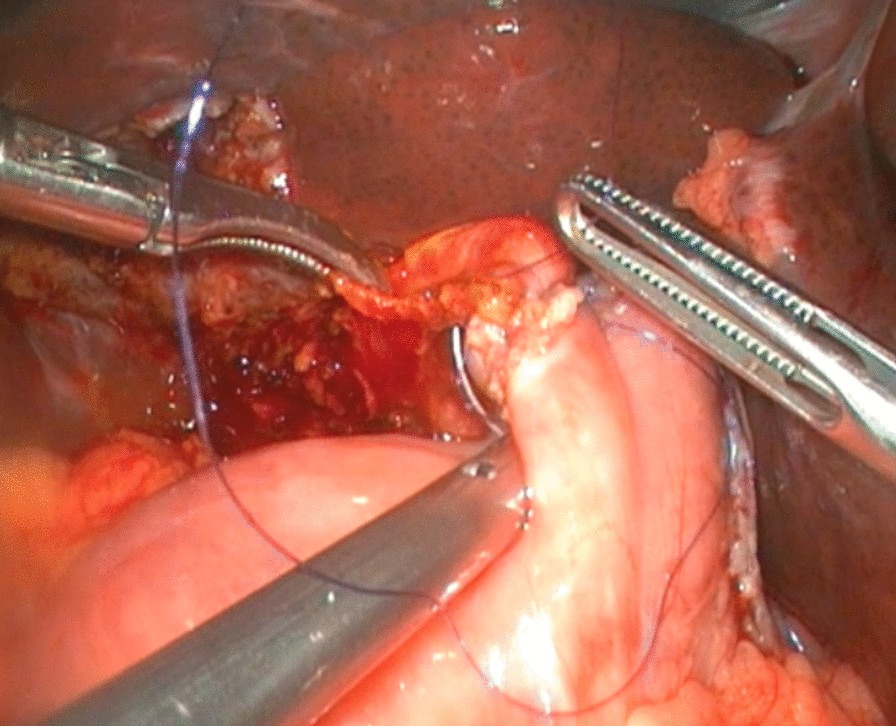
Posterior wall anastomosis during hepaticojejunostomy

**Fig.7:**
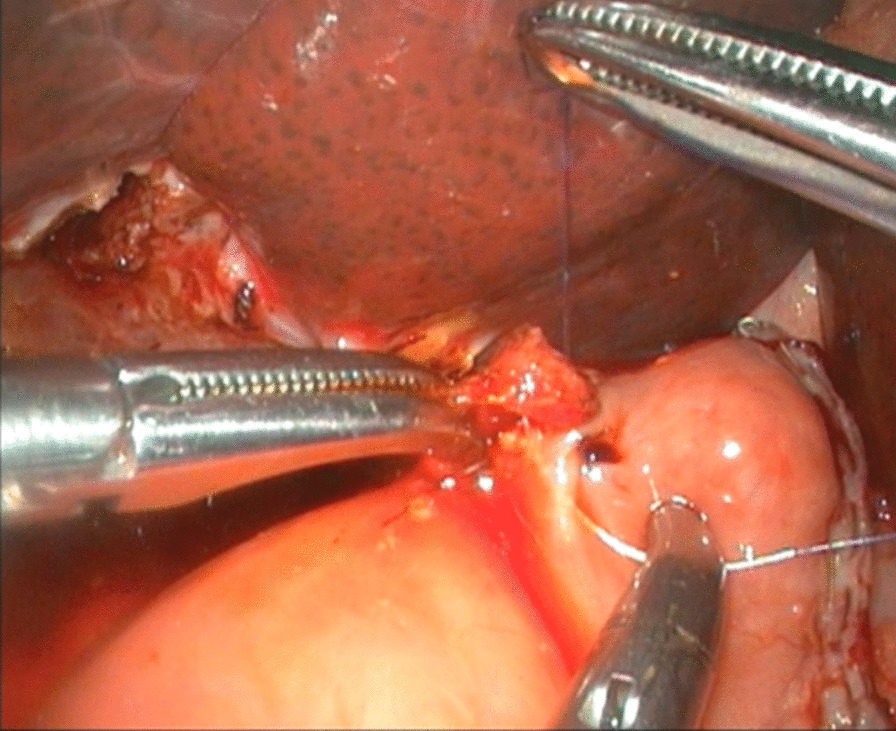
Anterior wall anastomosis during hepaticojejunostomy

**Fig.8:**
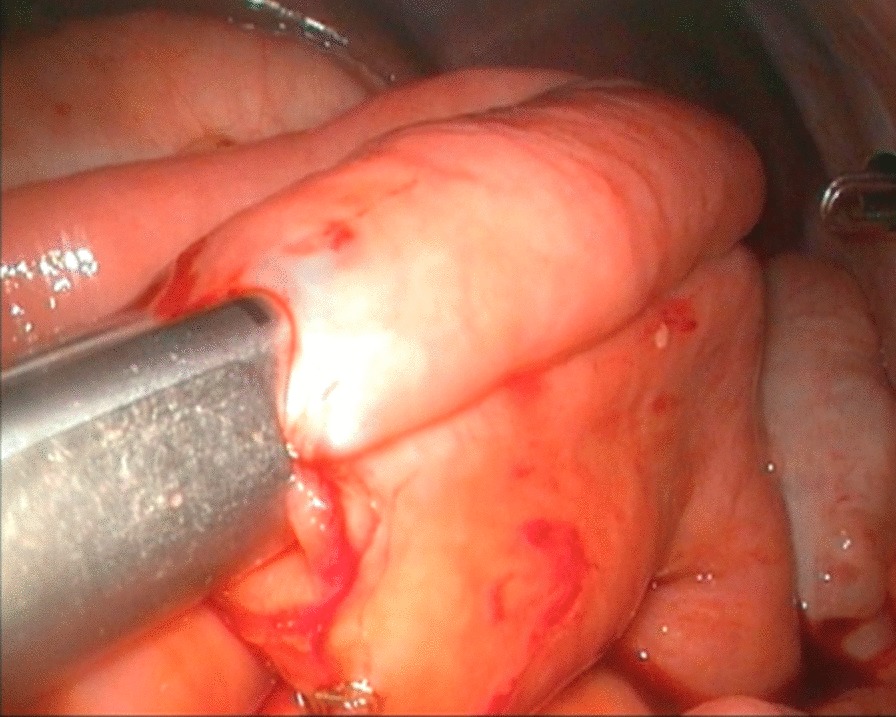
Jejunal side-to-side anastomosis with Endo-GIA tri-staple

**Fig.9:**
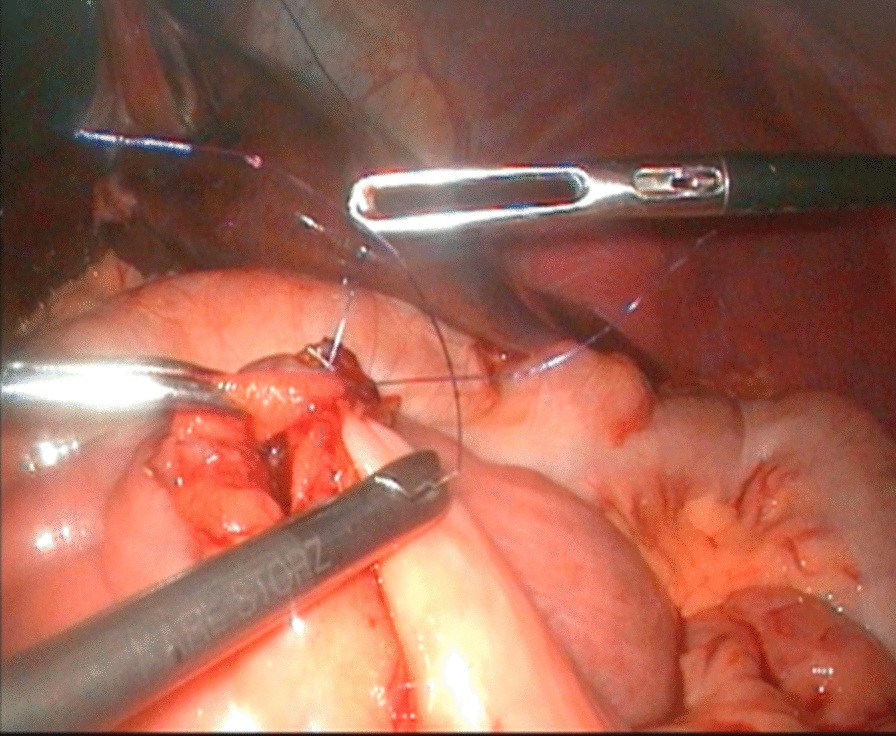
Suturing residual anastomosis

The ligament of Treitz was identified under laparoscopy, and the proximal jejunum 20 cm distal to the ligament was cut off using an Endo-GIA Tri-Staple instrument (COVIDIEN, USA) through the fourth port after extending the incision to 1.5–2.0 cm (Fig. 4). The jejunal mesentery was fully released (Fig. 5). To minimize the redundant Roux loop, an individualized jejunal Roux loop length was tailored by the distance between the umbilicus and the hepatic hilum [9]. The Roux loop was passed up to the hilum retrocolically. A side-hole jejunostomy was created on the antimesenteric border 1 cm from the end of the jejunal limb to match the diameter of the hepatic duct. End-to-side hepaticojejunal anastomosis was accomplished laparoscopically with continuous 5–0 or 6–0 PDS sutures (Figs. 6, 7). Then, the Endo-GIA Tri-Staple instrument was used to complete the jejunal side-to-side anastomosis (Fig. 8). The residual stoma was closed using continuous 5–0 PDS sutures, and the seromuscular layer was reinforced using intermittent 5–0 PDS sutures (Fig. 9).

#### Conventional laparoscopic CDC excision and Roux-en-Y hepaticojejunostomy

The trocar locations, cyst excision and Roux-en-Y hepaticojejunostomy were all consistent with those in the TLH group; the main difference was that the end-to-side jejunum-jejunum anastomosis was performed by extracorporeal manual sewing. The specific procedures were as follows: Firstly, identify the ligament of Treitz under laparoscopy. Secondly, capture the proximal jejunum 20 cm distal to the ligament with a bowel clamp, exteriorize it through the umbilical trocar site after extending the incision to 1.5 or 2.0 cm. Finally, carry out the Roux-en-Y end-to-side jejunojejunostomy with 2-layer interrupted sutures manually outside the abdomen and the closed jejunum limb was then lifted through an incision in the right side of the mesocolon to accomplish the end-to-side hepaticojejunal anastomosis.

Postoperatively, the patients were allowed to start oral intake when they met the following standards: (1) no abdominal distension; (2) light gastric juice color with a volume < 20 ml/day; and (3) patients had passage of flatus and recovery of bowel sounds. When the patients were afebrile and had no need for intravenous fluids, their wound was healing well, and their blood test results were normal, they were discharged.

### Follow up

Postoperative follow-ups were conducted to all patients 1, 3, 6 and 12 months since surgery completion and every 6 months thereafter. Each visit was constituted of physical examination, abdominal ultrasonography, and laboratory tests. Postoperative complications, for instance pancreatitis, anastomotic stenosis, intrahepatic stone formation and pancreatic calculi formation, were clinically evaluated matching with appropriate investigations.

### Statistical analysis

Data were analyzed with the SPSS 21.0 package. χ^2^ tests were performed to compare the distribution of sex, CDC type and postoperative complications between the TLH and CLH groups. Student’s *t-*tests were applied to compare the age, CDC length, CDC diameter, time to oral intake, postoperative hospital stay, hospitalization expenses, intraoperative blood loss and time for jejunum-cojejunum anastomosis. A level of *P* < 0.05 was considered statistically significant.

## Results

In our study, 55 patients (TLH = 30, CLH = 25) with a diagnosis of CDCs underwent cyst resection and end-to-side hepaticojejunostomy. There were no significant differences in the demographic variables or pathological manifestations of the cysts between the two groups (Table 1).

**Table 1:** Demographics of the TLH group vs the CLH group

TLH group (n = 30) CLH group (n = 25) *P*				
Sex (Male/female)		8/22	6/19	0.82^a^
Age (years)		4.31 ± 0.46 (1.3 years–12 years)	3.62 ± 0.50 (30 d-10 years)	0.30^b^
CDC Todani classification (I/IV)		16/14	19/6	0.08^a^
CDC length (mm)		46.47 ± 4.35	45.56 ± 3.59	0.88^b^
CDC diameter (mm)		28.67 ± 4.12	26.16 ± 2.67	0.63^b^

Values are mean ± standard deviation

^a^χ^2^ test

^b^Student *t* test

The postoperative hospital stay and time to oral intake in the TLH group were significantly shorter than those in the CLH group (5.50 ± 0.28 d vs 7.00 ± 0.74, *P* < 0.05; 3.57 ± 0.19 vs 4.56 ± 0.27, *P* < 0.01). It is worth noting that the hospitalization cost in the TLH group was significantly higher than that in the CLH group (CNY 40,085 ± 2447 vs CNY 26,084 ± 2776, *P* < 0.001). There were no significant differences in the intraoperative blood loss volume, time for jejunum-cojejunum anastomosis or complications between the two groups (Table 2).

**Table 2:** Perioperative characteristics of the TLH group vs the CLH group

	TLH group (n = 30)	CLH group (n = 25)	*P*
Time to oral intake (days)	3.57 ± 0.19	4.56 ± 0.27	<0.01^b^
Postoperative hospital stay (days)	5.50 ± 0.28	7.00 ± 0.74	<0.05^b^
Hospitalization expenses (CNY)	40,085 ± 2447	26,084 ± 2776	<0.001^b^
Intraoperative blood loss (ml)	9.57 ± 3.28	8.20 ± 1.13	0.72^b^
Time for jejunum-co-jejunum anastomosis (min)	80.50 ± 2.46	75.00 ± 2.04	0.10^b^
Postoperative complications (%)	3/30	2/25	0.79^a^

Values are mean ± standard deviation

^a^χ^2^ test

^b^Student *t* test

The median follow-up periods of the TLH and CLH groups were 17 (11–27 months) and 16 (11–27 months) months, respectively. All patients, except for those mentioned below, were free of abdominal pain, fever and jaundice. Group TLH had 3 postoperative complications. One patient experienced obstruction and necrosis 1 week after discharge (2 weeks after the operation) because the distal biliary jejunum herniated from the transverse mesocolic hiatus into the colon and compressed the proximal biliary jejunum, and choledochojejunostomy was performed again. One patient presented adhesive intestinal obstruction 1 month after discharge (1 month after surgery), which improved after conservative treatment. One patient underwent choledochojejunostomy again 1 month after discharge (1 month after surgery) because of severe adhesion in the abdominal cavity due to suppurative appendicitis and obstruction necrosis caused by cord compression of the proximal biliary jejunum.

Group CLH had 2 postoperative complications. One patient presented with an intestinal obstruction 1 week after the operation, and no relief was achieved after conservative treatment. This obstruction was found to be caused by umbilical incision and intestinal tube adhesion via a reoperation. One patient presented with fever, abdominal bloating, and increased leucocytes and amylase at 1 week after the operation, and ultrasonic examination indicated peritoneal effusion, which was not improved after conservative treatment. A pancreatic fistula was confirmed by surgery.

## Discussion

CDCs are a rare disorder of bile duct dilation that was first described by Vater and Ezler in 1723 [10]. Although cysts are benign lesions, they are closely related to many serious complications, such as malignant bile duct tumors, cholangitis, pancreatitis and intrahepatic bile duct stones [2, 11]. Therefore, surgery should be performed as soon as possible after the diagnosis is clear [12, 13]. Compared with traditional open surgery, laparoscopic CDC surgery has the following advantages [14, 15]: ① Laparoscopy can magnify the tissue 4–8 times, allowing precise separation of the cyst from the surrounding tissues, such as the hepatic artery, portal vein, pancreas and capillary network around the cyst, and thus avoiding side effects. ② Laparoscopy can penetrate into the hepatic hilum for a more accurate operation; meanwhile, bile duct abnormalities, such as labyrinthine bile duct and hepatic stenosis, can be visually detected. ③ Laparoscopic surgery causes less intestinal disturbance and allows faster postoperative intestinal peristalsis recovery. ④ The incision is small and aesthetic, and the pain is mild. The incidence of wound infection and incisional hernia is reduced after this operation. Total laparoscopic surgery, in addition to the above advantages, has an additional advantage in that the umbilical incision is smaller and more aesthetic, which is more satisfactory according to the needs of the children and their families.

In the CLH group, the bowel was pulled out through an umbilical incision to complete the jejunal end-to-side anastomosis. Intestinal traction and exposure increase the risk of intestinal injury and adhesions in theory. We successfully performed total laparoscopic CDC excision and Roux-en-Y hepaticojejunostomy in 30 children. Compared with those in the CLH group, the postoperative fasting time and hospitalization duration were significantly shorter in the TLH group. These findings show that the total laparoscopic approach disturbs the bowel less and allows faster recovery of gastrointestinal function. However, it is worth noting that the hospitalization cost in the TLH group was significantly higher than that in the CLH group, which may be due to the high cost of laparoscopic instruments. With the continuous development of surgical instruments, we believe that in the near future, the cost of laparoscopic instruments will gradually decrease.

The main difference between TLH and CLH is jejunum-to-jejunum anastomosis. We need to pay attention to the following points: ① When the jejunum is cut using an endoscopic stapler, the intestinal tube should be fully flattened to avoid overlap, which could result in insufficient cutting and an intestinal fistula. ② A side-hole jejunotomy was created on the antimesenteric border just 0.5 cm from the end of the proximal jejunum, to minimize the occurrence of a "blind pouch". ③ When using an endoscopic cutter stapler for jejunal side-to-side anastomosis, the puncture hole in the intestinal wall does not need to be large. Its size should be suitable for placement at the end of the stapler to minimize the residual stoma and reduce the operative duration. Additionally, side-to-side anastomosis of the jejunum should be arranged in parallel with the mesentery to ensure full contact with the stapler. In the early stages of implementing total laparoscopic surgery, there was one case in which a child underwent jejunal side-to-side anastomosis with an uneven arrangement, resulting in excessive residual anastomosis, increased suture difficulties and a prolonged operation. In this study, the time for jejunal side-to-side anastomosis in the TLH group was longer than the time for jejunal end-to-side anastomosis in the CLH group, but there was no significant difference. We consider that this finding may be related to the learning curve for mastering total laparoscopic surgery.

Postoperative complications of laparoscopic CDC excision include pancreatitis [16], pancreatic fistula [17], cholangitis [1], biliary fistula and intestinal obstruction [18]. In this study, one child in the TLH group required reoperation because the distal biliary jejunum herniated from the transverse mesocolic hiatus and compressed the proximal biliary loops, resulting in obstruction and necrosis. The transverse mesocolon and gastrocolic ligament are often thicker in older children with CDCs complicated with repeated infection; thus, we suggest that after establishing a retrocolic tunnel to cross the transverse mesocolon, the gastrocolic ligament should be fully separated at the same time so that the intestinal tube can pass smoothly. Subsequently, the hepatic limb jejunal and transverse mesocolon should be sutured intermittently with 3 to 4 needles to avoid obstruction caused by intestinal hernia. In the CLH group, one child developed a postoperative pancreatic fistula and needed reoperation to retain the abdominal drainage tube. We considered the pancreatic fistula to have been caused by too deep of an operating position when the distal end of the cyst was separated, resulting in damage to the pancreatic duct. Li et al. [19] believed that in children with cystic dilatation, not ligating the distal stump is a feasible approach and may minimize pancreatic duct injury.

The limitations of this study are that the number of cases is small and that the follow-up period is short. The long-term effect in the two groups needs further study.

## Conclusions

In summary, total laparoscopic CDC resection and Roux-en-Y hepaticojejunostomy are safe and feasible. Although the TLH operation was difficult because of the limited abdominal cavity capacity of children, resulting in a narrow operation space within delicate tissue, recovery was faster and the hospitalization duration was shorter in the TLH group than in the CLH group. Based on our experience with radical laparoscopic CDC excision and intestinal anastomosis with a stapler, tacit cooperation and precise operations can ensure the safety and efficacy of the operation. In addition, the hospitalization cost in the TLH group was relatively high, so surgical plans should be optimized for patients considering their family's economic situation.

## Data Availability

The datasets used and/or analysed during the current study are available from the corresponding author on reasonable request.

## Abbreviations

TLH: Total laparoscopic hepaticojejunostomy
CLH: Conventional laparoscopic hepaticojejunostomy
CDC: Choledochal cyst
CT: Computed tomography
MRCP: Magnetic resonance cholangiopancreatography
